# Paxalisib with Trastuzumab for HER2-Positive Metastatic Breast Cancer with Active Brain Metastases: A Phase II Trial with Integrated Plasma and CSF ctDNA Analyses

**DOI:** 10.1158/2767-9764.CRC-26-0571

**Published:** 2026-11-06

**Authors:** Elia Segui, Jorge Gomez Tejeda Zañudo, Noah Graham, Patrick T. Kurnia, Andrea N. Perry, Nabihah Tayob, Heather A. Parsons, Raechel Davis, Molly K. DiLullo, Jennifer A. Ligibel, Filipa Lynce, Jing Ni, Sara M. Tolaney, Eric P. Winer, Adrienne G. Waks, Jean Zhao, Rinath M. Jeselsohn, Nancy U. Lin, José P. Leone

**Affiliations:** 1 Medical Oncology, Dana-Farber Cancer Institute, Boston, Massachusetts.; 2 Harvard Medical School, Boston, Massachusetts.; 3 Translational Genomics and Targeted Therapeutics in Solid Tumors, August Pi i Sunyer Biomedical Research Institute (IDIBAPS), Barcelona, Spain.; 4 Broad Institute, Boston, Massachusetts.; 5 Department of Data Science, Dana-Farber Cancer Institute, Boston, Massachusetts.; 6 Department of Cancer Biology, Dana-Farber Cancer Institute, Boston, Massachusetts.; 7 Yale Cancer Center, New Haven, Connecticut.

## Abstract

**Purpose:**

Human epidermal growth factor receptor 2 (HER2)–positive breast cancer brain metastases (BCBM) frequently harbor PI3K/AKT/mTOR alterations. Preclinical HER2+ BCBM models demonstrated durable tumor regression with dual PI3K and mTOR inhibition. Our phase II trial evaluated paxalisib, a brain-penetrant dual PI3K/mTOR inhibitor, combined with trastuzumab.

**Patients and Methods:**

Eligible patients had active/progressive HER2+ BCBM and could have received prior therapies; leptomeningeal disease was excluded. The trial included a safety run-in to determine the recommended phase II dose (RP2D), followed by expansion cohorts. The primary endpoint was confirmed central nervous system (CNS) objective response rate (ORR) per Response Assessment in Neuro-Oncology Brain Metastases (RANO-BM) criteria at the RP2D. Paired plasma and cerebrospinal fluid (CSF) circulating tumor DNA (ctDNA) was analyzed using ultralow-pass whole-genome sequencing (ULP-WGS) and whole-exome sequencing (WES).

**Results:**

Seventeen patients were treated (median age, 44 years; median 5 prior therapy lines). Paxalisib 30 mg daily plus trastuzumab was established as the RP2D. Among 12 patients treated at the RP2D, no confirmed CNS responses occurred [CNS ORR, 0%; 95% confidence interval (CI), 0%–26.5%], and the study closed for futility. The median overall survival (OS) was 16.5 months. Treatment was feasible, with a safety profile consistent with PI3K/mTOR inhibition. Exploratory ctDNA analyses showed higher tumor fraction (TF) in CSF than plasma, with longitudinal changes in CSF TF paralleling CNS tumor burden.

**Conclusions:**

Paxalisib combined with trastuzumab was feasible but did not meet the primary endpoint of CNS ORR in heavily pretreated HER2+ BCBM. Exploratory findings suggest that CSF-derived ctDNA may better capture tumor genomics and treatment response than plasma ctDNA in HER2+ BCBM.

**Significance:**

The brain-penetrant dual PI3K/mTOR inhibitor paxalisib plus trastuzumab showed minimal activity in heavily pretreated HER2+ BCBM. Paired liquid biopsy analyses demonstrated that CSF-derived ctDNA may better reflect tumor genomics and treatment response than plasma, supporting CSF for translational studies in this setting.

**Trial Registration:**

ClinicalTrials.gov identifier: NCT03765983.

## Introduction

Human epidermal growth factor receptor 2-positive (HER2+) breast cancer, defined by the overexpression of HER2, accounts for approximately 15% to 20% of breast cancers (3). Along with triple-negative breast cancer, HER2+ tumors have the highest rates of breast cancer brain metastases (BCBM), with up to 30% to 50% of patients developing central nervous system (CNS) involvement (4, 5). Local treatments, including surgery, whole-brain radiotherapy (WBRT), and stereotactic radiosurgery (SRS), can be beneficial; however, they do not address extracranial progression, and further CNS progression typically occurs within 6 to 12 months (6–8). Moreover, as survival improves in patients with HER2+ BCBM (9), the cognitive deterioration associated with WBRT has become an increasing concern (10).

Advances in systemic therapies have significantly improved outcomes for HER2+ metastatic breast cancer, with HER2-targeted therapies remaining the mainstay of treatment (11, 12). Although trastuzumab deruxtecan (T-DXd), tucatinib, neratinib, and ado-trastuzumab emtansine (T-DM1) are active in patients with HER2+ BCBM (13–15), nearly all patients eventually experience disease progression, commonly in the CNS. Therefore, more effective systemic therapies for HER2+ BCBM are needed.

Targeting the PI3K/AKT/mTOR pathway is emerging as a promising strategy in breast cancer (16–20). Approximately 30% to 40% of HER2+ tumors harbor PI3K/AKT/mTOR alterations (21, 22), with *PIK3CA* mutations and *PTEN* loss linked to resistance to anti-HER2 therapies (23, 24) and worse prognosis (25). Beyond driving therapy resistance, PI3K pathway activation, particularly via *PTEN* loss, plays a key role in HER2+ BCBM, supporting PI3K/mTOR inhibition as a potential therapeutic approach in this setting (26–28). In preclinical models, including patient-derived xenografts (PDX) of HER2+ BCBM, HER2-targeted therapies combined with either α-selective or pan-PI3K inhibitors showed limited activity. However, simultaneous inhibition of PI3K and mTOR using separate agents led to significant and durable tumor regression, independent of *PIK3CA* mutation status (29). These findings provided the rationale for evaluating simultaneous PI3K and mTOR inhibition as a therapeutic strategy in HER2+ BCBM.

Paxalisib (GDC-0084), a potent, brain-penetrant dual inhibitor of class I PI3K and mTOR, was selected for this trial as a single-agent approach to achieve simultaneous inhibition of both pathways. Paxalisib was specifically designed to achieve high CNS exposure, thereby maximizing its ability to target brain cancers, including glioblastoma (GBM) and brain metastases from solid tumors (30). Prior clinical data in GBM demonstrated promising activity and a safety profile consistent with other PI3K/mTOR inhibitors (31).

Here, we report the final results of a phase II trial (NCT03765983) evaluating paxalisib (GDC-0084) in combination with trastuzumab in patients with active or progressive HER2+ BCBM together with correlative analyses of cerebrospinal fluid (CSF) and plasma circulating tumor DNA (ctDNA).

## Patients and Methods

### Patient population

Both male and female patients were eligible. Eligible patients had stage IV, HER2+ invasive breast cancer, defined according to the current American Society of Clinical Oncology/College of American Pathologists guidelines (32). The study had two cohorts. Cohort A enrolled patients with at least one measurable CNS metastasis (≥10 mm) and unequivocal radiographic evidence of new and/or progressive brain metastases. Cohort B was specifically designed for patients with new and/or progressive brain metastases who had a clinical indication for surgical resection, with resection planned between cycle 1 days 3 and 9 after the initiation of study therapy. Additional eligibility criteria included ≥18 years of age, Eastern Cooperative Oncology Group performance status ≤2, left ventricular ejection fraction ≥50%, and a stable or decreasing corticosteroid dose for at least 7 days prior to treatment initiation. There were no restrictions on the number of prior systemic therapies. Patients were excluded if they had received prior treatment with a PI3K or an mTOR inhibitor. Additional exclusion criteria included visceral crisis, CNS complications requiring neurosurgical intervention, uncontrolled diabetes, and known leptomeningeal disease.

### Study design and treatment

This was an open-label, nonrandomized, phase II study conducted at Dana-Farber Cancer Institute (Boston, MA, USA; Clinicaltrials.gov identifier: NCT03765983). Patients and investigators were not blinded to treatment. The complete study protocol is available in the Supplementary Methods.

The trial was designed to include an initial 6-patient safety run-in (cohort A safety run-in) to evaluate dose-limiting toxicities (DLT), followed, if no safety signals were identified, by two expansion cohorts treated at the recommended phase II dose (RP2D). Expansion cohorts included cohort A, a single-arm, two-stage phase II cohort, and cohort B, a presurgical window cohort.

The initial dose for the safety run-in consisted of paxalisib 45 mg orally once daily in combination with trastuzumab administered intravenously at an 8 mg/kg loading dose followed by 6 mg/kg every 21 days. This dose selection was informed by prior clinical studies of paxalisib monotherapy in GBM, in which 45 mg once daily was shown to be feasible and well tolerated and was established as the maximum tolerated dose (31). If one or fewer DLTs were observed, this dose would be declared the RP2D. If two or more DLTs occurred, the paxalisib dose would be reduced to 30 mg orally once daily with the same trastuzumab regimen. If one or fewer DLTs were observed at the reduced dose, it would be declared the RP2D; otherwise, study enrollment would be discontinued, and the study would close.

DLTs were defined as clinically significant treatment-related grade 3 adverse events (AE), or grade ≥2 nonhematologic toxicities requiring dose reduction, occurring during the first 21-day treatment cycle and graded according to the National Cancer Institute Common Terminology Criteria for Adverse Events (NCI CTCAE) version 5.0. Toxicities attributable to disease progression, intercurrent illness, or concomitant medications were excluded. Detailed DLT definitions are provided in the study protocol.

All treatment was administered on an outpatient basis. Radiographic assessments were performed every two cycles (approximately every 6 weeks) for the first four cycles and every three cycles (approximately every 9 weeks) thereafter. CNS disease response and progression were evaluated using Response Assessment in Neuro-Oncology Brain Metastases (RANO-BM) criteria (33), and extracranial disease was assessed using RECIST v1.1. Patients remained on study until CNS progression per RANO-BM, extracranial progression per RECIST v1.1 (34), clinical progression at the investigator’s discretion, unacceptable toxicity, or other protocol-defined discontinuation criteria. After treatment discontinuation, patients were followed for CNS progression, if not already observed, and overall survival (OS).

This study was approved by the Dana-Farber/Harvard Cancer Center Institutional Review Board and conducted in accordance with the International Conference on Harmonization Good Clinical Practice guidelines and the Declaration of Helsinki. Written informed consent was obtained from all patients prior to study participation.

### Statistical design and analysis

The primary endpoint was confirmed objective response rate (ORR) in the CNS per RANO-BM criteria among patients treated at the RP2D in cohort A. The planned primary endpoint for cohort B was to correlate pharmacodynamic inhibition of p-4EBP1 in resected brain tumor tissue from patients exposed to paxalisib with an intracranial response to paxalisib plus trastuzumab in the corresponding PDX models generated from the same resected tumors. Secondary endpoints included safety and tolerability of the combination; clinical benefit rate (CBR) at 18 and 24 weeks; duration of response; progression-free survival (PFS); extracranial ORR per RECIST v1.1; and OS.

The efficacy analysis population included all patients who initiated protocol therapy at the RP2D, and all efficacy endpoints were assessed in this population unless otherwise specified. The safety population included all patients who received at least one dose of study treatment and had at least one postbaseline safety assessment; AEs were graded according to NCI CTCAE version 5.0.

Cohort A followed a Simon two-stage design with a planned enrollment of 37 patients. In the first stage, 12 patients were to be enrolled at the RP2D; if at least one CNS response was observed, accrual would proceed to the second stage enrolling an additional 25 patients. The regimen was considered worthy of further study if at least four responses were observed among 37 patients, providing 90% power with a one-sided type I error rate of 10%. Cohort B was planned to enroll 10 patients.

Descriptive statistics were used to summarize safety and efficacy endpoints. Time-to-event endpoints, including bicompartmental PFS per RANO-BM criteria, single-compartment PFS per RECIST v1.1 criteria, and OS, were estimated using the Kaplan–Meier method with 90% confidence intervals (CI). Bicompartmental PFS was defined as the time from the first dose of paxalisib (cycle 1 day 1) to disease progression (CNS or body) or death from any cause, whichever occurred first; patients alive without disease progression were censored at the date of last disease evaluation. OS was defined as the time from the first dose of paxalisib (cycle 1 day 1) to death from any cause; patients without death were censored at the date they were last known to be alive.

All analyses were conducted using the Statistical Analysis System (RRID: SCR_008567), version 9.4, and R Project for Statistical Computing (RRID: SCR_001905), version 4.3.1.

### Correlative analyses of CSF and plasma ctDNA

ctDNA analyses were performed using plasma and CSF samples prospectively collected during the study. CSF collection was mandated only during the safety run-in phase and could be waived if deemed contraindicated. CSF samples (5–10 mL) were collected by standard lumbar puncture at baseline and at cycle 2 day 1 (±7 days). Blood samples were collected longitudinally at prespecified on-treatment time points (baseline, cycle 2 day 1, and cycle 3 day 1) and at disease progression or study discontinuation.

ctDNA extracted from CSF and plasma was analyzed using ultralow-pass whole-genome sequencing (ULP-WGS) to estimate tumor fraction (TF). Whole-exome sequencing (WES) was performed in samples with sufficient total ctDNA material and TF ≥10%, with all cases having TF ≥25% for this study. Correlative analyses aimed to characterize the genomic landscape of HER2+ BCBM, evaluate clonal architecture and phylogenetic relationships over time, compare genomic profiles derived from CSF and plasma, and explore associations between alterations in the PI3K/AKT/mTOR pathway and clinical outcomes. In addition, analyses assessed whether baseline or on-treatment TF in CSF or plasma was associated with clinical outcomes.

Detailed descriptions of sequencing procedures, data processing, variant calling, copy-number analysis, and evolutionary modeling are provided in the Supplementary Methods.

## Results

### Patient characteristics

A total of 17 patients were enrolled to cohort A and initiated treatment between February 2019 and March 2024. No patients were enrolled to cohort B. Baseline characteristics are summarized in Table 1, and study representativeness is detailed in Supplementary Table S1. The median age was 44 years (range, 33–61), and all patients were female. All tumors were HER2+, and 67% were hormone receptor–positive.

**Table 1.** Baseline patient and treatment characteristics (*n* = 17).

Characteristic	Number of patients (%)
Age, years	
Median (range)	44.3 (33.5–60.6)
Sex	
Female	17 (100%)
Race	
Asian	1 (5.9%)
Black/African American	3 (17.6%)
White	12 (70.6%)
Other	1 (5.9%)
Ethnicity	
Non-Hispanic	17 (100%)
ECOG PS at baseline	
0	10 (58.8%)
1	6 (35.3%)
2	1 (5.9%)
Stage at initial diagnosis	
I–III	10 (58.8%)
IV	7 (41.2%)
HR status at metastatic diagnosis	
Negative	5 (33.3%)
Positive	10 (66.7%)
Unknown	2 (11.8%)
Disease sites at trial enrollment^a^	​
CNS	17 (100%)
Other visceral	6 (35.3%)
Liver	4 (23.5%)
Lung	2 (11.8%)
Bone	13 (76.5%)
Breast/chest wall	3 (17.6%)
Regional lymph nodes	2 (11.8%)
Lymph nodes, other	3 (17.6%)
Prior therapy for metastatic disease	
CNS surgery	5 (33.3%)
WBRT	6 (35.3%)
SRS	12 (70.6%)
Prior lines of systemic therapy^b^, median (range)	5 (1–10)

Abbreviations: CNS, central nervous system; ECOG PS, Eastern Cooperative Oncology Group performance status; HR, hormone receptor; SRS, stereotactic radiosurgery; WBRT, whole-brain radiotherapy.

a Patients could have involvement at more than one disease site at trial enrollment.

b Prior lines refer to systemic therapies administered in the metastatic setting, including HER2-targeted therapies (monoclonal antibodies and tyrosine kinase inhibitors), endocrine therapy, chemotherapy, and antibody–drug conjugates.

All patients had CNS involvement at study entry, per eligibility criteria. In addition, most patients had bone metastases (77%), and 35% had other visceral diseases, including liver and/or lung involvement. All patients had received prior HER2-directed therapy for metastatic breast cancer, with a median of 5 prior lines of therapy (range, 1–10). Local therapy directly to the CNS included neurosurgery (29%), WBRT (35%), and SRS (71%).

### Safety run-in and determination of the RP2D

The trial incorporated an initial safety run-in to evaluate DLTs and establish the RP2D of paxalisib in combination with trastuzumab. Five patients were treated at the starting dose of paxalisib 45 mg daily (dose level 1). Two DLTs were observed during the first treatment cycle. One patient experienced grade 2 anorexia, fatigue, and diarrhea requiring dose reduction. The second developed grade 3 nausea with associated grade 2 vomiting and anorexia, resulting in grade 3 dehydration and electrolyte abnormalities (hyponatremia, hypokalemia, and hypomagnesemia), also requiring dose reduction. These events led to discontinuation of enrollment at this dose level.

**Figure 1.**
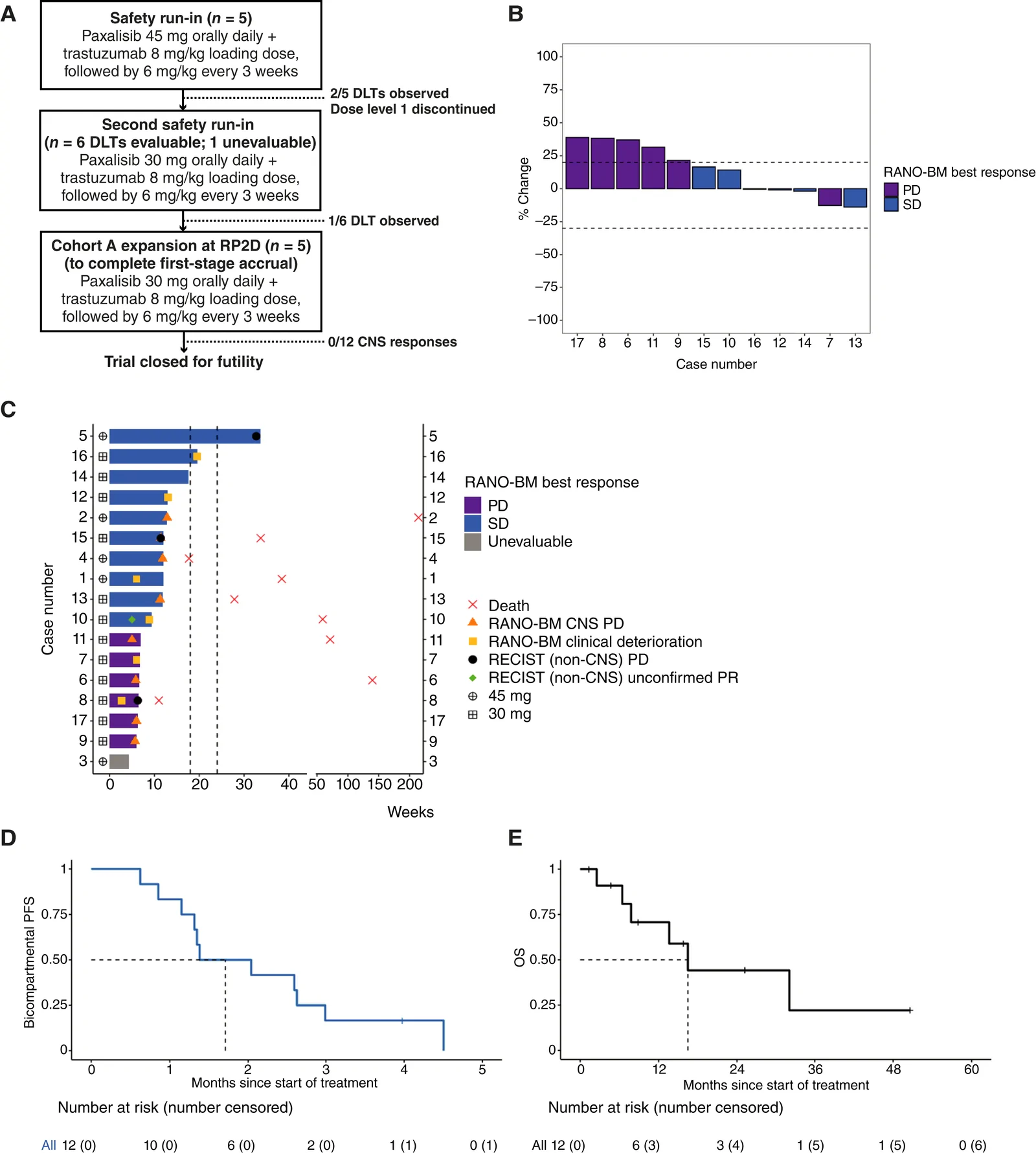
Study flow, antitumor activity, and survival outcomes. **A,** Safety run-in, RP2D determination, and study flow. The study incorporated an initial safety run-in to evaluate DLTs and establish the RP2D of paxalisib in combination with trastuzumab. Five patients were treated at dose level 1 (paxalisib 45 mg daily), with two DLTs observed, leading to discontinuation of this dose level. A second safety run-in was conducted at dose level −1 (paxalisib 30 mg daily), in which six patients were evaluable for DLT assessment, and one DLT was observed. Paxalisib 30 mg daily was declared the RP2D and selected for expansion in cohort A. Twelve patients were treated at the RP2D in the first stage of cohort A, with no confirmed CNS responses per RANO-BM criteria. The trial was subsequently closed for futility. DLTs were assessed during the first 21-day treatment cycle. One patient in the second safety run-in was unevaluable for DLT assessment; therefore, one additional patient was enrolled to replace this patient. **B,** Best change in CNS target lesions. Waterfall plot showing the best percentage change from baseline in CNS target lesions assessed by RANO-BM among patients in the primary analysis population treated at the RP2D (*n* = 12). Case 7 was classified as progressive disease (PD) per RANO-BM due to clinical deterioration. **C,** Treatment duration and response. Swimmer plot depicting treatment duration (weeks) among all treated patients (*n* = 17). Each bar represents an individual patient and is colored according to best overall response per RANO-BM. Symbols indicate key clinical events, including CNS progression per RANO-BM, extracranial progression per RECIST v1.1, clinical deterioration, unconfirmed partial response (PR) per RECIST v1.1, and death, as indicated in the legend. Circles denote the paxalisib 45 mg cohort and triangles the 30 mg cohort. Case 2 met criteria for RANO-BM progressive disease; however, treatment discontinuation was due to toxicity. Case 7 was classified as progressive disease based on clinical deterioration, although treatment was discontinued because of toxicity. One additional patient discontinued treatment for clinical progression (case 16). **D,** Bicompartmental PFS. Kaplan–Meier estimate of bicompartmental PFS among patients treated at the RP2D in the primary analysis population (*n* = 12). **E,** OS. Kaplan–Meier estimate of OS among patients treated at the RP2D in the primary analysis population (*n* = 12).

Based on these findings, a second safety run-in was conducted at a reduced paxalisib dose of 30 mg daily (dose level −1) to further characterize safety. Six patients were evaluable for DLT assessment at this dose level; one additional patient was replaced because a grade 3 diarrhea event was considered unevaluable for DLT determination, due to delayed initiation of supportive care. One DLT was observed, consisting of grade 4 cytokine release syndrome. No additional DLTs occurred. Accordingly, paxalisib 30 mg daily in combination with trastuzumab was declared the RP2D and selected for expansion cohorts (Fig. 1A).

### Safety and tolerability

Most patients treated at the RP2D experienced at least one treatment-related adverse event (TRAE) of grade ≥2 (83%, 10/12 patients). Grade 3 TRAEs occurred in 33% of patients and grade 4 TRAEs in 8%; no grade 5 toxicities were observed.

The most common TRAEs of any grade were fatigue (42%), diarrhea (25%), hyperglycemia (25%), oral mucositis (17%), maculopapular rash (17%), and generalized muscle weakness (17%; Table 2). The most frequent grade ≥3 TRAE was diarrhea (17%). A single grade 4 event of cytokine release syndrome was observed.

**Table 2.** TRAEs among patients treated at RP2D (paxalisib 30 mg; *n* = 12).

Treatment-related adverse event (TRAE)	All grades, *n* (%)	Grade 2, *n* (%)	Grade 3, *n* (%)	Grade 4, *n* (%)
Any event	10 (83.3)	5 (41.7)	4 (33.3)	1 (8.3)
Fatigue	5 (41.7)	5 (41.7)	​	​
Diarrhea	3 (25)	1 (8.3)	**2 (16.7)**	​
Hyperglycemia	3 (25)	2 (16.7)	1 (8.3)	​
Generalized muscle weakness	2 (16.7)	1 (8.3)	1 (8.3)	​
Mucositis oral	2 (16.7)	2 (16.7)	​	​
Rash maculopapular	2 (16.7)	1 (8.3)	1 (8.3)	​
Abdominal pain	1 (8.3)	1 (8.3)	​	​
Alanine aminotransferase increased	1 (8.3)	1 (8.3)	​	​
Anorexia	1 (8.3)	1 (8.3)	​	​
Aspartate aminotransferase increased	1 (8.3)	1 (8.3)	​	​
Cytokine release syndrome	1 (8.3)	​	​	1 (8.3)
Dehydration	1 (8.3)	​	1 (8.3)	​
Dyspnea	1 (8.3)	1 (8.3)	​	​
Hoarseness	1 (8.3)	1 (8.3)	​	​
Hyponatremia	1 (8.3)	​	1 (8.3)	​
Hypophosphatemia	1 (8.3)	​	1 (8.3)	​
Hypoxia	1 (8.3)	​	1 (8.3)	​
Nausea	1 (8.3)	1 (8.3)	​	​
Pain	1 (8.3)	1 (8.3)	​	​
Pneumonitis	1 (8.3)	1 (8.3)	​	​

NOTE: Grade 1 toxicities were not collected. No grade 5 toxicities were observed. All TRAEs are shown. Grade 3 events occurring in ≥10% of patients are shown in bold.

Overall, 58% of patients treated at the RP2D required at least one paxalisib dose hold, 8% required dose reduction, and 8% discontinued treatment because of toxicity.

Safety outcomes from the initial 45 mg safety run-in cohort (*n* = 5) are provided in Supplementary Table S2.

### Efficacy

Among patients treated in the safety run-in cohort at 45 mg (*n* = 5), four patients achieved stable disease (SD) as the best overall response per RANO-BM criteria, and one patient was unevaluable. The best percentage change in CNS target lesions per RANO-BM criteria among all treated patients (*n* = 17) is shown in Supplementary Fig. S1.

Among the 12 patients treated at the RP2D in the first stage of cohort A, no confirmed CNS responses were observed, corresponding to a CNS ORR of 0% (95% CI, 0%–26.5%; Table 3; Fig. 1B). As the prespecified criterion to proceed to the second stage was not met, the trial was closed for futility, and no further enrollment occurred in cohort A or cohort B.

**Table 3.** Efficacy outcomes by RANO-BM and RECIST v1.1 in the primary analysis population^a^ treated at paxalisib 30 mg (*n* = 12).

Endpoint	All patients treated at 30 mg (*n* = 12)
RANO-BM	
ORR, *n* (%; 95% CI)	0 (0%, 0–26.5)
CBR^b^ at 18 weeks, *n* (%; 95% CI)	1 (8.3%, 0.2–38.5)
CBR^b^ at 24 weeks, *n* (%; 95% CI)	0 (0%, 0–26.5)
Best overall response, *n* (%)	​
SD	6 (50%)
SD ≥ 18 weeks	1 (16.7%)
SD ≥ 24 weeks	0 (0%)
PD	6 (50%)
RECIST v1.1	
ORR, *n* (%; 95% CI)	0 (0%, 0–26.5)
CBR^b^ at 18 weeks, *n* (%; 95% CI)	0 (0%, 0–26.5)
CBR^b^ at 24 weeks, *n* (%; 95% CI)	0 (0%, 0–26.5)
Best overall response, *n* (%)	​
Unconfirmed PR^c^	1 (8.3%)
SD/non-CR/non-PD	8 (66.7%)
SD/non-CR/non-PD ≥ 18 weeks	0 (0%)
SD/non-CR/non-PD ≥ 24 weeks	0 (0%)
PD	1 (8.3%)
Unevaluable	2 (16.7%)
Time-to-event outcomes	
Median bicompartmental PFS^d^, months (95% CI)	1.71 (1.32–NE)
Median PFS per RECIST 1.1, months (95% CI)	NR (2.7–NE)
Median OS, months (95% CI)	16.5 (7.8–NE)
Follow-up	
Median follow-up, months (IQR)	25.2 (8.9–50.6)

Abbreviations: CR, complete response; IQR, interquartile range; NE, not estimable; NR, not reached; PD, progressive disease; PR, partial response.

a Primary analysis population includes patients treated at the 30 mg dose level.

b CBR was defined as CR, PR, or SD lasting ≥18 or ≥24 weeks, as specified.

c Unconfirmed PR reflects response not meeting confirmation criteria per RECIST v1.1.

d Bicompartmental PFS was defined as the time from treatment initiation to progression per either RANO-BM or RECIST v1.1, or death from any cause.

In the RP2D cohort, six (50%) patients achieved SD as the best overall response per RANO-BM criteria, although only one patient maintained SD for ≥18 weeks, resulting in an 18-week CBR of 8.3% (95% CI, 0.2%–38.5%; Table 3; Fig. 1C). The remaining six (50%) patients had progressive disease as the best overall response. One additional patient in the safety run-in cohort maintained SD for ≥24 weeks (Fig. 1C).

The median survival follow-up was 25.2 months. Among the 12 patients treated at the RP2D, the median bicompartmental PFS was 1.71 months [95% CI, 1.32–not reached (NR); Fig. 1D]. Disease progression was predominantly CNS driven, with radiographic RANO-BM progression observed in five patients, RANO-BM–defined clinical deterioration in five patients, and concurrent CNS and extracranial progression per RANO-BM and RECIST v1.1 in one patient. The median OS was 16.5 months (95% CI, 7.8–NR; Fig. 1E). At the time of data cutoff (June 2024), no patients remained on study treatment.

### CSF and plasma ctDNA analyses

Among the 17 treated patients, ctDNA analyses were performed in eight patients with available samples. ULP-WGS was conducted on paired baseline and cycle 2 day 1 (±7 days) plasma samples from eight patients and paired CSF samples from five patients. WES was performed in samples with sufficient ctDNA content, comprising 10 samples (7 CSF and 3 plasma) from 5 patients, including longitudinal samples from 4 patients (3 CSF pairs and 1 plasma pair; Supplementary Fig. S2).

#### Genomic landscape of CSF and plasma ctDNA

**Figure 2.**
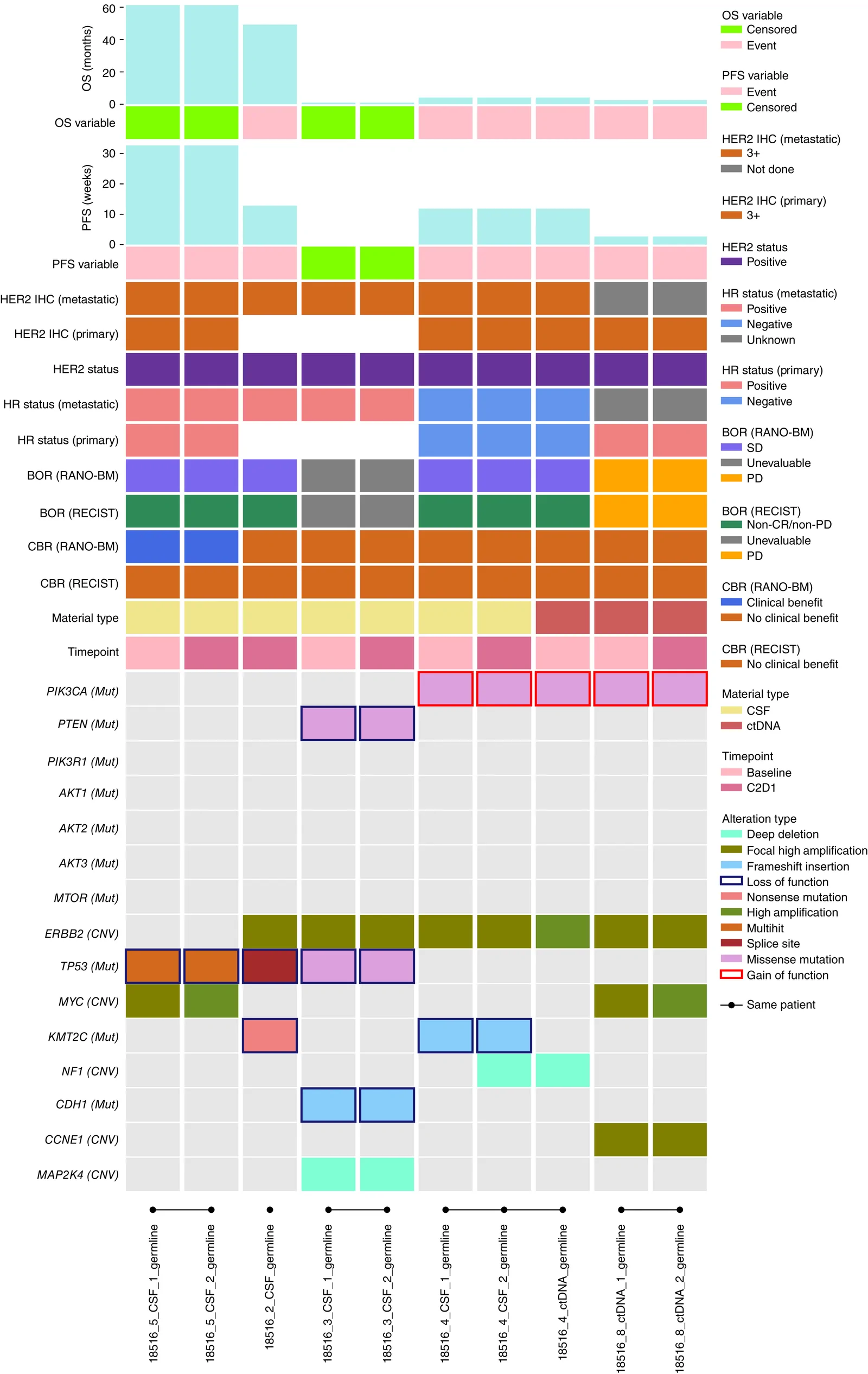
Genomic landscape of CSF and plasma ctDNA by WES (*n* = 10 samples/5 patients). Oncoprint summarizing genomic alterations detected by WES in CSF and ctDNA samples from five patients included in the correlative analyses (10 total samples: 7 CSF and 3 plasma samples). Columns represent individual samples, grouped by patient, and rows depict selected clinicopathologic features and recurrent genomic alterations relevant to HER2+ breast cancer. Genomic alterations include single-nucleotide variants and copy-number gains or losses, as indicated in the legend. Clinical annotations include hormone receptor (HR) status, HER2 status, best overall response assessed by RANO-BM and RECIST (v1.1), CBR, PFS, and OS status. Sample timing [baseline and on-treatment at cycle 2 day 1 (C2D1)] is indicated. BOR, best objective response; CNV, copy-number variation; CR, complete response.

Among the five patients with available WES data, alterations commonly observed in HER2+ metastatic breast cancer were identified. *ERBB2* amplification was detected in four of five patients, and somatic oncogenic mutations detected included *TP53* (3/5), *PIK3CA* (2/5), and *PTEN* (1/5). *MYC* and other proliferation-related high-level copy-number alterations, including *CCNE1*, were observed in three of five patients. Notably, the only patient who derived prolonged clinical benefit (RANO-BM SD >24 weeks) lacked detectable alterations in the PIK3CA pathway (Fig. 2).

#### TF dynamics

**Figure 3.**
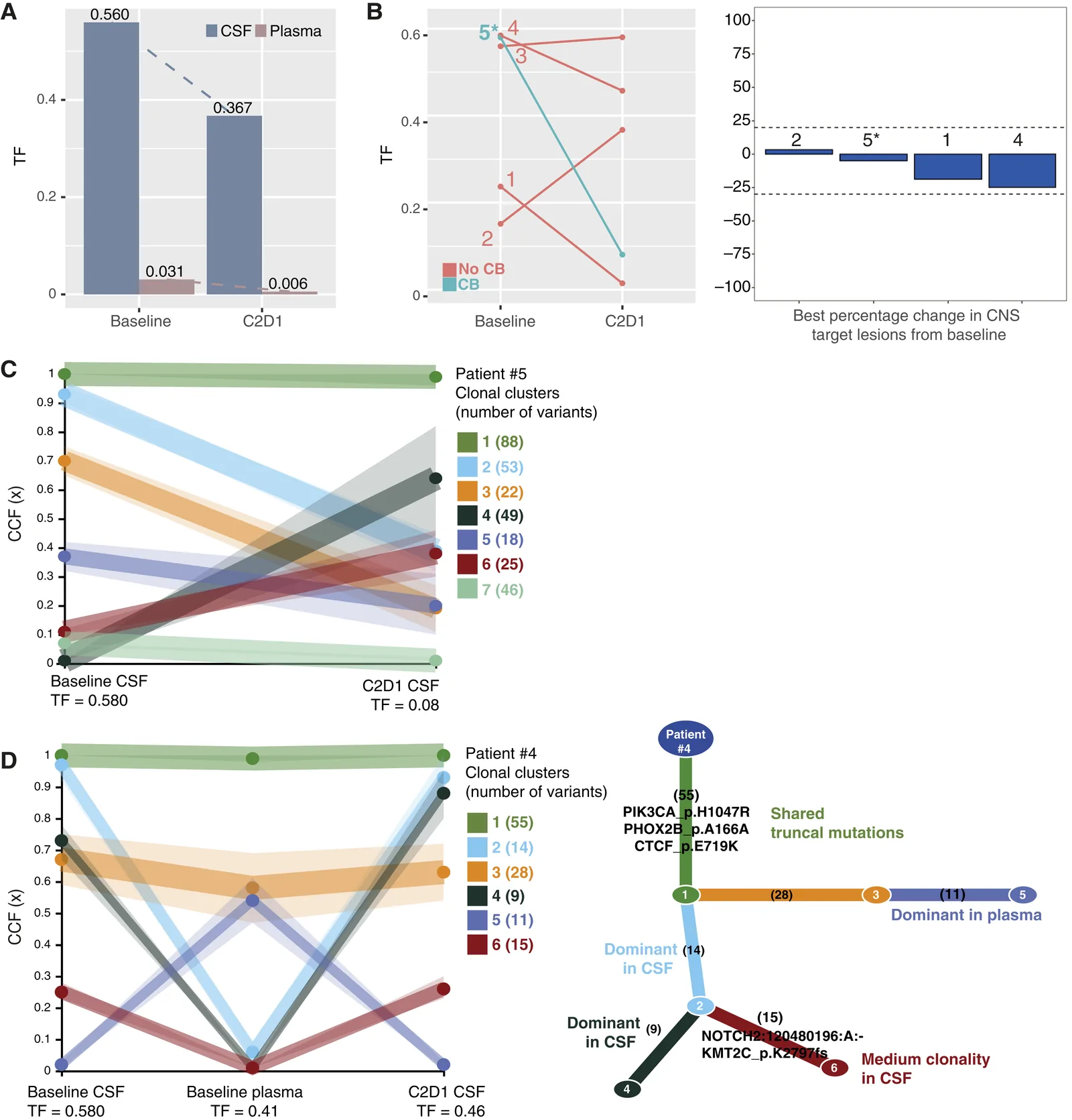
TF dynamics, clonal evolution, and phylogenetic analyses from plasma and CSF and plasma ctDNA. **A,** Median TF estimated by ULP-WGS in paired plasma and CSF samples at baseline and cycle 2 day 1 (C2D1). TF was consistently higher in CSF than plasma at both timepoints (*n* = 10 samples/5 patients). **B,** Longitudinal CSF TF dynamics in individual patients relative to changes in CNS target lesions assessed by RANO-BM criteria (*n* = 10 samples/5 patients). Patients with declining CSF TF exhibited reductions in CNS tumor burden, whereas increasing TF was associated with lesion growth or nonevaluable disease. The asterisk (*) identifies the patient who derived clinical benefit. **C,** Clonal evolution in CSF ctDNA from patient #5, showing changes in cancer cell fraction (|ΔCCF| >0.20) between timepoints. **D,** Phylogenetic reconstruction for patient #4 based on WES of plasma and CSF ctDNA, illustrating shared truncal mutations and CSF subclones present at low clonality in plasma. CB, clinical benefit.

Using ULP-WGS, ctDNA TF was longitudinally assessed in paired plasma and CSF samples. ctDNA TF was consistently higher in CSF than in plasma, both at baseline (median TF, 0.560 vs. 0.031) and on treatment at cycle 2 day 1 (median TF, 0.367 vs. 0.006; Fig. 3A). Longitudinal changes in CSF TF generally paralleled changes in CNS target lesions: Three of five patients with declining CSF TF exhibited tumor shrinkage, although none met RANO-BM criteria for partial response, whereas two patients with increasing CSF TF demonstrated lesion growth or were unevaluable. Notably, the patient with prolonged clinical benefit showed the most pronounced CSF TF decline, from 0.58 at baseline to 0.08 on treatment (Fig. 3B). In contrast, changes in plasma TF did not consistently parallel changes in CNS target lesions; for example, patient #8 had CNS progression per RANO-BM despite a decline in plasma TF (Supplementary Fig. S3).

#### Clonal evolution and phylogenetics

Analysis of longitudinal WES data revealed evidence of clonal evolution in two of four patients, defined by changes in cancer cell fraction (|ΔCCF| > 0.20), including the patient with prolonged clinical benefit, who exhibited the most pronounced clonal dynamics over time (Fig. 3C). Phylogenetic analysis in one patient identified shared truncal mutations in both plasma and CSF, together with additional CSF-specific subclones present at low clonality in plasma (Fig. 3D).

## Discussion

Activation of the PI3K/AKT/mTOR pathway represents a critical mechanism of resistance to anti-HER2 therapies (23, 24) and plays an important role in the biology of HER2+ BCBM (26–29). Despite strong preclinical evidence showing that dual inhibition of PI3K and mTOR yields durable tumor regression in PDX models of HER2+ BCBM (29), this phase II study demonstrated limited clinical activity of paxalisib plus trastuzumab in a heavily pretreated population with active or progressive HER2+ BCBM. No confirmed CNS responses were observed among the 12 patients treated at the RP2D (CNS ORR, 0%; 95% CI, 0%–26.5%), and the trial was discontinued for futility after completion of the first stage of accrual. These findings underscore the challenges of translating promising preclinical combination strategies into meaningful clinical benefit in advanced CNS disease.

Despite the lack of clinical activity, the combination of paxalisib and trastuzumab was feasible, with a safety profile consistent with known class effects of PI3K/mTOR inhibitors (16–19). The most frequent TRAEs included fatigue, diarrhea, hyperglycemia, and oral mucositis; grade 4 events were uncommon and consisted of hyperglycemia and cytokine release syndrome. Tolerability improved at the RP2D of paxalisib 30 mg daily, supporting the feasibility of further investigation of brain-penetrant PI3K/mTOR inhibitors in alternative combination strategies.

Several factors may explain the limited clinical activity observed despite compelling preclinical data. Preclinical studies demonstrated that HER2-targeted therapies combined with either α-selective or pan-PI3K inhibitors alone had limited activity and that robust and durable tumor regression required dual inhibition of pan-PI3K and mTOR using two distinct agents (29). Notably, buparlisib (BKM120), the pan-PI3K inhibitor used in key preclinical studies, was no longer being actively developed in breast cancer at the time this trial was designed (35). Paxalisib was therefore selected as a brain-penetrant dual PI3K/mTOR inhibitor with the goal of achieving simultaneous inhibition of both pathways while mitigating the overlapping toxicities associated with combining separate pan-PI3K and mTOR inhibitors, which have been challenging to coadminister at full doses in prior clinical studies (36). However, this strategy may have resulted in insufficient pathway inhibition in a heavily pretreated population with advanced CNS disease in which greater pathway suppression may be required to achieve meaningful clinical activity.

In addition, this phase II trial evaluated concurrent HER2 and PI3K/mTOR pathway inhibition in the absence of a chemotherapy backbone. In contrast, prior studies incorporating mTOR inhibition in HER2+ BCBM generally included chemotherapy and reported greater clinical activity. In a phase II study of everolimus combined with trastuzumab and vinorelbine, intracranial response rates were low; however, approximately one third of patients achieved sustained CNS clinical benefit at 6 months despite being heavily pretreated (37). Similarly, the TRIO-US B-09 phase Ib/II study evaluating everolimus, lapatinib, and capecitabine reported a CNS ORR of 27% at 12 weeks and a median PFS of 6.2 months (38). In this context, the clinical activity observed with the chemotherapy-free paxalisib–trastuzumab doublet was modest, suggesting that dual PI3K/mTOR inhibition combined with trastuzumab alone may be insufficient in this setting and that future therapeutic strategies may require additional systemic partners, including chemotherapy-based approaches, to achieve meaningful clinical benefit in HER2+ BCBM.

Despite the modest clinical activity observed, the median OS of 16.5 months from study entry provides important clinical context in this heavily pretreated HER2+ BCBM population, a setting for which prospective clinical data remain limited. Patients enrolled in this study had received a median of five prior lines of systemic therapy, representing a more heavily pretreated population than those typically enrolled in most prospective HER2+ BCBM trials. Although contemporary HER2-directed therapies with CNS activity, including T-DXd and tucatinib-based regimens, have substantially improved outcomes for patients with HER2+ BCBM (14, 15), many patients ultimately develop progressive CNS disease and continue to have limited treatment options. As survival improves, patients increasingly experience repeated CNS progression and cumulative toxicities from local therapies, highlighting the continued need for effective brain-penetrant systemic therapies capable of overcoming resistance mechanisms within the CNS.

A major strength of this study is the prospective collection of paired baseline and on-treatment plasma and CSF samples for ctDNA analyses. Although brain metastases represent a major clinical challenge in metastatic breast cancer, their genomic characterization remains limited because of restricted access to tumor tissue (39). Plasma ctDNA has limited sensitivity for CNS disease, whereas CSF-derived ctDNA may better represent the genomic landscape of brain metastases, even in the absence of leptomeningeal involvement (40, 41). This study provided an opportunity to explore the potential utility of CSF-derived ctDNA for molecular characterization and longitudinal assessment of CNS disease.

In this exploratory analysis, WES of plasma- and CSF-derived ctDNA identified alterations commonly observed in HER2+ metastatic breast cancer (21), including frequent *TP53* oncogenic mutations, *MYC* amplifications, and other proliferation-related amplifications, in line with aggressive disease biology. Using ULP-WGS, ctDNA TF was consistently higher in CSF than in plasma and longitudinal changes in CSF TF generally paralleled changes in CNS tumor burden, whereas plasma TF dynamics did not show a consistent association with CNS target lesion changes. Although clonal evolution was limited in most samples, the patient with prolonged clinical benefit exhibited the most pronounced clonal dynamics over time. In addition, phylogenetic analysis in one patient identified shared truncal mutations in both plasma and CSF, alongside CSF-specific subclones detected at low clonality in plasma. Although based on a limited number of samples, these descriptive findings are consistent with the potential utility of CSF-derived ctDNA for capturing tumor genomics and longitudinal changes in CNS disease and warrant validation in larger prospective studies. There are several limitations to this study. First, the small sample size limits the precision of the efficacy estimates and the ability to draw broader conclusions about clinical activity. However, enrollment was terminated according to the prespecified two-stage design after no confirmed CNS responses were observed in the first stage at RP2D. In addition, the heavily pretreated nature of the study population may have limited the ability to detect meaningful clinical activity. At the same time, this reflects a clinically relevant population with few effective treatment options that remains underrepresented in prospective interventional studies. Although preclinical studies supported a chemotherapy-free backbone, it remains uncertain whether greater activity might have been observed had the study been designed in combination with cytotoxic therapy; however, an important consideration is the toxicity already observed in the absence of chemotherapy. From a translational perspective, the limited number of available plasma and CSF biospecimens, particularly those with sufficient TF for WES, restricted the scope of genomic and evolutionary analyses. As a result, the correlative findings should be considered exploratory and hypothesis generating.

In conclusion, although treatment options for patients with HER2+ BCBM have expanded substantially in recent years, more effective systemic therapies remain needed. This phase II study demonstrates that the combination of the dual PI3K/mTOR inhibitor paxalisib with trastuzumab is feasible but shows minimal clinical activity in a heavily pretreated population with active or progressive HER2+ BCBM. The observed OS together with the integrated translational analyses provides important clinical and biological context for future therapeutic development in HER2+ BCBM. Importantly, the prospective collection and analysis of CSF-derived ctDNA highlight its potential utility for capturing tumor genomics and longitudinal changes in CNS disease, supporting further evaluation of CSF-based biomarkers in larger prospective studies.

## Supplementary Material

Supplementary Figure S1 Best percentage change in CNS target lesions from baseline per RANO-BM criteria among all treated patients (n=17).

Supplementary Figure S2 REMARK diagram for CSF and plasma ctDNA analyses.

Supplementary Figure S3 Longitudinal plasma tumor fraction (TF) dynamics in individual patients relative to changes in CNS target lesions assessed by RANO-BM criteria.

Supplementary Methods Supplementary Methods

Supplementary Table S1 Representativeness of Study Participants

Supplementary Table S2 Treatment-related adverse events (TRAEs) among patients treated at 45 mg safety run-in (n=5)

## Data Availability

The data that support the findings of this study are available from the corresponding author upon reasonable request. Data requests will be reviewed by the corresponding author to determine whether the request is subject to any intellectual property or confidentiality obligations. Data sharing may require a data/material transfer agreement.
