# Supplementary material for: Paxalisib with Trastuzumab for HER2-Positive Metastatic Breast Cancer with Active Brain Metastases: A Phase II Trial with Integrated Plasma and CSF ctDNA Analyses
Source: Cancer Res Commun. 2026 Nov 6;6(11):2619–30. doi: 10.1158/2767-9764.CRC-26-0571 (PMC13741795; doi:10.1158/2767-9764.CRC-26-0571)
Supplement: Supplementary file 1 [file crc-26-0571_supplementary_figure_s1_suppsf1.docx]

**Supplementary Fig. S1. Best percentage change in CNS target lesions from baseline per RANO-BM criteria among all treated patients (n=17).** Bars represent individual patients and are colored according to best overall CNS response. One patient was unevaluable for RANO-BM response, and is omitted.

**Abbreviations:** CNS, central nervous system; RANO-BM, Response Assessment in Neuro-Oncology Brain Metastases.
