# Supplementary material for: Paxalisib with Trastuzumab for HER2-Positive Metastatic Breast Cancer with Active Brain Metastases: A Phase II Trial with Integrated Plasma and CSF ctDNA Analyses
Source: Cancer Res Commun. 2026 Nov 6;6(11):2619–30. doi: 10.1158/2767-9764.CRC-26-0571 (PMC13741795; doi:10.1158/2767-9764.CRC-26-0571)
Supplement: Supplementary file 2 [file crc-26-0571_supplementary_figure_s2_suppsf2.docx]

**Supplementary Fig. S2. REMARK diagram for CSF and plasma ctDNA analyses.** Flow diagram summarizing patient inclusion and sample availability for circulating tumor DNA (ctDNA) analyses. Among 17 treated patients, ctDNA analyses were performed in 8 patients with available samples. Ultra–low-pass whole-genome sequencing (ULP-WGS) was performed in paired baseline and cycle 2 day 1 (±7 days) plasma samples from 8 patients and paired CSF samples from 5 patients. Whole-exome sequencing (WES) was performed in samples with sufficient ctDNA content, comprising 10 samples (7 CSF and 3 plasma) from 5 patients.

^a^ Paired samples refer to baseline and cycle 2 day 1 (±7 days) collections; ^b^ CSF collection was mandated only during the 45mg safety run-in phase (n=5) and could be waived if deemed contraindicated; ^c^ WES was performed only in samples meeting predefined ctDNA content criteria (TF ≥10%).

**Abbreviations:** CSF, cerebrospinal fluid
