# Supplementary material for: Paxalisib with Trastuzumab for HER2-Positive Metastatic Breast Cancer with Active Brain Metastases: A Phase II Trial with Integrated Plasma and CSF ctDNA Analyses
Source: Cancer Res Commun. 2026 Nov 6;6(11):2619–30. doi: 10.1158/2767-9764.CRC-26-0571 (PMC13741795; doi:10.1158/2767-9764.CRC-26-0571)
Supplement: Supplementary file 3 [file crc-26-0571_supplementary_figure_s3_suppsf3.docx]

**Supplementary Fig. S3.**

Longitudinal plasma tumor fraction (TF) dynamics in individual patients relative to changes in CNS target lesions assessed by RANO-BM criteria. Plasma TF was measured at baseline and cycle 2 day 1 (C2D1). Lines represent individual patients and are colored according to best overall CNS response per RANO-BM criteria.

**Abbreviations:** TF, tumor fraction; CNS, central nervous system; BOR, best objective response; RANO-BM, Response Assessment in Neuro-Oncology Brain Metastases; C2D1, cycle 2 day 1; PD, progressive disease; SD, stable disease.
