# Supplementary material for: Paxalisib with Trastuzumab for HER2-Positive Metastatic Breast Cancer with Active Brain Metastases: A Phase II Trial with Integrated Plasma and CSF ctDNA Analyses
Source: Cancer Res Commun. 2026 Nov 6;6(11):2619–30. doi: 10.1158/2767-9764.CRC-26-0571 (PMC13741795; doi:10.1158/2767-9764.CRC-26-0571)
Supplement: Supplementary file 4 [file crc-26-0571_supplementary_methods_suppsm.docx]

**Supplementary Methods**

Some of the methods reported follow closely what has been described in our recent work.^1-3^

*DNA extraction from plasma and cerebrospinal fluid (CSF) for cfDNA*

cfDNA was extracted from plasma or CSF using the QIA symphony DSP Circulating DNA Kit according to the manufacturer’s instructions, with 6.3 mL as input and with a 60 all DNA elution (Qiagen, 2017).

*Library Construction for cfDNA*

Initial DNA input is normalized to be within the range of 25-52.5 ng in 50 all of TE buffer (10mM Tris HCl 1mM EDTA, pH 8.0) according to picogreen quantification. Library preparation is performed using a commercially available kit provided by KAPA Biosystems (KAPA HyperPrep Kit with Library Amplification product KK8504) and IDT’s duplex UMI adapters. Unique 8-base dual index sequences embedded within the p5 and p7 primers (purchased from IDT) are added during polymerase chain reaction (PCR). Enzymatic clean-ups are performed using Beckman Coultier AMPure XP beads with elution volumes reduced to 30μL to maximize library concentration.

*Preparation of libraries for cluster amplification and sequencing for cfDNA*

Post Library Construction Quantification and Normalization. Library quantification was performed using the Invitrogen Quant-It broad range dsDNA quantification assay kit (Thermo Scientific Catalog: Q33130) with a 1:200 PicoGreen dilution. Following quantification, each library is normalized to a concentration of 35 ng/μL, usingTris-HCl, 10mM, pH 8.0.

Library Pool Creation for Ultra-low Pass Whole Genomic Sequencing (ULP-WGS). In preparation for the sequencing of the ultra-low pass libraries (ULP), approximately, 4 μL of the normalized library is transferred into a new receptacle and further normalized to a concentration of 2ng/μL using Tris-HCl, 10mM, pH 8.0. Following normalization, up to 95 ultra-low pass WGS samples are pooled together using equivolume pooling. The pool is quantified via quantitative PCR (qPCR) and normalized to the appropriate concentration to proceed to sequencing.

In-solution hybrid selection for whole-exome sequencing (WES). After library construction, hybridization and capture are performed using the relevant components of IDT’s XGen hybridization and wash kit and following the manufacturer’s suggested protocol, with several exceptions. A set of 12-plex pre-hybridization pools are created. These pre-hybridization pools are created by equivolume pooling of the normalized libraries, Human Cot-1 and IDT XGen blocking oligos. The pre-hybridization pools undergo lyophilization using the Biotage SPE-DRY. Post lyophilization, custom exome bait (TWIST Biosciences) along with hybridization mastermix is added to the lyophilized pool prior to resuspension. Samples are incubated overnight. Library normalization and hybridization setup are performed on a Hamilton Starlet liquid handling platform, while target capture is performed on the Agilent Bravo automated platform. Post capture, a PCR is performed to amplify the capture material.

After post-capture enrichment, library pools are quantified using qPCR (automated assay on the Agilent Bravo), using a kit purchased from KAPA Biosystems with probes specific to the ends of the adapters. Based on qPCR quantification, pools are normalized using a Hamilton Starlet to 2nM.

*Cluster amplification and sequencing for cfDNA*

ULP-WGS. Cluster amplification of library pools was performed according to the manufacturer’s protocol (Illumina) using Exclusion Amplification cluster chemistry and NovaSeq SP flowcells. Flowcells were sequenced on a NovaSeq 6000 Sequencer using the XP workflow and a v1.5 300 cycle NovaSeq SP kit. Each pool of ultra-low pass whole genome libraries is run on one lane using paired 151bp runs.

WES. Cluster amplification of library pools was performed according to the manufacturer’s protocol (Illumina) using Exclusion Amplification cluster chemistry and HiSeq X flowcells. Flowcells were sequenced on v2 Sequencing-by-Synthesis chemistry for HiSeq X flowcells. The flowcells are then analyzed using RTA v.2.7.3 or later. Each pool of libraries was run on paired 151bp runs, reading the dual-indexed sequences to identify molecular indices and sequenced across the number of lanes needed to meet coverage for all libraries in the pool.

*Ultra Low Pass Whole Genome Sequence data processing and tumor fraction estimation*

Cell-free DNA (cfDNA) isolated from plasma and cerebrospinal fluid samples underwent ultra–low-pass whole-genome sequencing (ULP-WGS) to estimate tumor fraction. Sequencing libraries were prepared using Illumina-compatible workflows and sequenced at low genome-wide coverage. Reads were aligned to the GRCh37 (hg19) human reference genome. Tumor fraction (TFx) was estimated using ichorCNA.^4^ Tumor fraction estimates were used to assess tumor DNA content and to determine eligibility for downstream WES analyses.

*WES data processing and analysis*

Data processing and quality control. WES was performed on cfDNA samples with sufficient total ctDNA material and TFx≥10% based on ULP-WGS analysis, with all cases having TFx ≥25% for this study. Whole exome sequences for each sample were captured using Illumina technology and the sequencing data processing and analysis was performed using the Picard and Terra pipelines at the Broad Institute. The Picard pipeline (http://picard.sourceforge.net) was used to produce a BAM file with aligned reads. This includes alignment to the GRCh37 human reference sequence using the Burrows-Wheeler Aligner (BWA)^5^ and estimation and recalibration of base quality score with the Genome Analysis Toolkit (GATK).^6^

A custom-made cancer genomics analysis pipeline was used to identify somatic alterations using the Terra platform (<https://app.terra.bio/>). The Cancer Genome Analysis (CGA)

WES Characterization pipeline developed at the Broad Institute was used to call, filter and annotate somatic mutations and copy number variation (available in the Terra platform public workspace broad-fc-getzlab-workflows/CGA_WES_Characterization_OpenAccess) (documentation available in <https://docs.google.com/document/d/1VO2kX_fgfUd0x3mBS9NjLUWGZu794WbTepBel3cBg08>). The pipeline employs the following tools: MuTect,^7^ ContEst,^8^ Strelka2,^9^ Orientation Bias Filter,^10^ DeTiN,^11^ AllelicCapSeg,^12^ MAFPoNFilter,^13^ BLAT realignment filter,^14^ ABSOLUTE,^15^ GATK,^6^ GATK CNV,^16^ Picard Tools CrosscheckFingerprints and CollectMultipleMetrics (<https://broadinstitute.github.io/picard/>), Variant Effect Predictor,^17^ and Oncotator.^18^

GATK CNV^16^ was used for the generation of accurate relative copy- number profiles from the whole exome sequencing data and reference/alternate read counts at heterozygous SNP sites present in both the normal and tumor samples. After accurate proportional coverage profiles are generated for a sample, Allelic CapSeg tool^12^ was used to generate a segmented allelic copy ratio profile. Allelic copy number profile and mutational call data were modeled jointly by ABSOLUTE^15^ to produce purity for the samples, a discrete copy number profile, and compute cancer cell fractions (CCF).

All tumors (and paired normal samples for sequencing for applicable QC methods) used for downstream analysis passed coverage QC (% target bases at 50x > 80% or mean target coverage > 200), cross-sample contamination QC (contamination<5% from GATK CalculateContamination or ContEst), sample-mixup QC (Picard CrosscheckFingerprints LOD score>0), tumor-in-normal QC (tumor-in-normal level<0.25 from DeTiN), and had a purity of 10% or more.

*Annotation of somatic alterations*

The OncoKB^19^ annotator was used (<https://github.com/oncokb/oncokb-annotator>) to annotate known oncogenic mutations, identify their effect (e.g. loss of function or gain of function), and if they are known cancer hotspots. For the comutation plots, silent, introns, UTRs, and RNA variants are not included (i.e., we remove those classified as "Silent", "Intron", "3'UTR", "3'Flank", "5'Flank", "5'UTR", "IGR", "lincRNA", or "RNA"). Variants included in the comutation plot that are not in a hotspot region and not assigned an oncogenic effect by OncoKB are classified as either missense (if classified as a “Missense_Mutation”), putative loss of function if classified as either of these mutations: "Splice_Site","Frame_Shift_Del", "Frame_Shift_Ins", "Nonsense_Mutation", "Nonstop_Mutation", "In_Frame_Del", "Start_Codon_SNP","In_Frame_Ins", "Start_Codon_Ins", "Stop_Codon_Del", or "Stop_Codon_Ins"), or other mutation. The multi-hit classification is used if there are multiple alterations in the gene (excluding silent or intron variants).

*Copy number alterations and biallelic inactivation*

The inference of gene amplifications, gene deletions, and biallelic inactivations were based on the copy number profile obtained from ABSOLUTE.^15^ To infer biallelic inactivations, mutational events that included both loss of heterozygosity (LOH) and a loss-of-function mutation (LOF) were used. Gene amplifications and deep deletions were based on the purity corrected measure for the segment containing that gene. Genes in a segment- specific copy number of less than 0.5 were considered deep deletions (Deep DEL). To measure segment-specific copy number amplifications, the genome ploidy was subtracted for each sample to obtain the copy number above ploidy (CNAP). CNAPs of at least 3 are considered as amplifications (AMP); CNAPs above 1.5, but below 3 are considered low amplification (GAIN); CNAPs of at least 6 are considered high amplifications (High AMP), and CNAPs of at least 9 and no more than 100 genes are considered focal high amplification (Focal High AMP). For the comutation plots, only AMPs, High AMPs, Focal High AMPs, and Deep DELs are included.

*Tumor evolutionary and phylogenetic analysis*

Patients with >1 tumor or ctDNA WES samples (including CSF samples) collected from different timepoints/locations were used to study tumor evolution and tumor heterogeneity. To properly compare SNVs and indels in samples from the same patient, the union of all mutations called in each patient’s samples were considered. The reference and alternate reads in each patient’s samples were used as input for ABSOLUTE^15^ to compute cancer cell fractions. The clonal structure and the evolutionary history of the clones (phylogenic tree) was inferred with PhylogicNDT [bioRxiv 508127; doi: <https://doi.org/10.1101/508127>] using SNVs and CNVs.

**Supplementary Methods References:**

1.	Jain E, Zañudo JGT, McGillicuddy M, et al: The Metastatic Breast Cancer Project: leveraging patient-partnered research to expand the clinical and genomic landscape of metastatic breast cancer and accelerate discoveries. medRxiv:2023.06.07.23291117, 2023

2.	Gómez Tejeda Zañudo J, Barroso-Sousa R, Jain E, et al: Exemestane plus everolimus and palbociclib in metastatic breast cancer: clinical response and genomic/transcriptomic determinants of resistance in a phase I/II trial. Nature Communications 15:2446, 2024

3.	Wander SA, Cohen O, Gong X, et al: The Genomic Landscape of Intrinsic and Acquired Resistance to Cyclin-Dependent Kinase 4/6 Inhibitors in Patients with Hormone Receptor-Positive Metastatic Breast Cancer. Cancer Discov 10:1174-1193, 2020

4.	Adalsteinsson VA, Ha G, Freeman SS, et al: Scalable whole-exome sequencing of cell-free DNA reveals high concordance with metastatic tumors. Nat Commun 8:1324, 2017

5.	Li H, Durbin R: Fast and accurate short read alignment with Burrows-Wheeler transform. Bioinformatics 25:1754-60, 2009

6.	McKenna A, Hanna M, Banks E, et al: The Genome Analysis Toolkit: a MapReduce framework for analyzing next-generation DNA sequencing data. Genome Res 20:1297-303, 2010

7.	Cibulskis K, Lawrence MS, Carter SL, et al: Sensitive detection of somatic point mutations in impure and heterogeneous cancer samples. Nat Biotechnol 31:213-9, 2013

8.	Cibulskis K, McKenna A, Fennell T, et al: ContEst: estimating cross-contamination of human samples in next-generation sequencing data. Bioinformatics 27:2601-2, 2011

9.	Kim S, Scheffler K, Halpern AL, et al: Strelka2: fast and accurate calling of germline and somatic variants. Nat Methods 15:591-594, 2018

10.	Costello M, Pugh TJ, Fennell TJ, et al: Discovery and characterization of artifactual mutations in deep coverage targeted capture sequencing data due to oxidative DNA damage during sample preparation. Nucleic Acids Res 41:e67, 2013

11.	Taylor-Weiner A, Stewart C, Giordano T, et al: DeTiN: overcoming tumor-in-normal contamination. Nat Methods 15:531-534, 2018

12.	Landau DA, Carter SL, Stojanov P, et al: Evolution and impact of subclonal mutations in chronic lymphocytic leukemia. Cell 152:714-26, 2013

13.	Lawrence MS, Stojanov P, Mermel CH, et al: Discovery and saturation analysis of cancer genes across 21 tumour types. Nature 505:495-501, 2014

14.	Kent WJ: BLAT--the BLAST-like alignment tool. Genome Res 12:656-64, 2002

15.	Carter SL, Cibulskis K, Helman E, et al: Absolute quantification of somatic DNA alterations in human cancer. Nat Biotechnol 30:413-21, 2012

16.	Auwera GAV, O'Connor BD: Genomics in the Cloud: Using Docker, GATK, and WDL in Terra. O'Reilly Media, 2020

17.	Shamsani J, Kazakoff SH, Armean IM, et al: A plugin for the Ensembl Variant Effect Predictor that uses MaxEntScan to predict variant spliceogenicity. Bioinformatics 35:2315-2317, 2019

18.	Ramos AH, Lichtenstein L, Gupta M, et al: Oncotator: cancer variant annotation tool. Hum Mutat 36:E2423-9, 2015

19.	Chakravarty D, Gao J, Phillips SM, et al: OncoKB: A Precision Oncology Knowledge Base. JCO Precis Oncol 2017, 2017
