# Supplementary material for: Paxalisib with Trastuzumab for HER2-Positive Metastatic Breast Cancer with Active Brain Metastases: A Phase II Trial with Integrated Plasma and CSF ctDNA Analyses
Source: Cancer Res Commun. 2026 Nov 6;6(11):2619–30. doi: 10.1158/2767-9764.CRC-26-0571 (PMC13741795; doi:10.1158/2767-9764.CRC-26-0571)
Supplement: Supplementary file 5 [file crc-26-0571_supplementary_table_s1_suppst1.docx]

**Supplementary Table S1.** Representativeness of Study Participants

| Cancer type(s)/ subtype(s)/stage(s)/condition | Human epidermal growth factor receptor 2-positive (HER2+) metastatic breast cancer (Stage IV) with active breast cancer brain metastases (BCBM) |
| --- | --- |
| **Considerations related to:** | |
| Sex | HER2+ breast cancer, similar to other breast cancer subtypes, is a predominantly female disease. Male breast cancer accounts for approximately 0.5–1% of all breast cancer diagnoses. Although HER2+ tumors represent about 15-20% of breast cancers overall and a comparable proportion of male breast cancers, the absolute number of affected men remains very small. Consequently, HER2+ metastatic breast cancer with brain metastases in men is rare. The absence of male participants in this study reflects the low incidence of this condition rather than an explicit exclusion criterion. |
| Age | HER2+ breast cancer is typically diagnosed at a younger age than HR+/HER2- disease, with a median age in the mid-to-late 50s in population-based datasets. In contemporary metastatic trials, including those enrolling patients with brain metastases, median ages are generally in the early-to-mid 50s. The median age in this study was 44 years (range, 33–61), which is younger than that reported in large phase III trials such as DESTINY-Breast12. This difference likely reflects the small sample size, referral patterns to a tertiary academic center, and eligibility criteria favoring patients with preserved performance status. |
| Race/ethnicity | The study population included patients self-identifying as Asian (5.9%), Black/African American (17.6%), White (70.6%), and Other (5.9%). The proportion of Black participants (17.6%) is comparable to or higher than that reported in many early-phase oncology and contemporary HER2+ metastatic trials. However, given the small sample size and single-center design, the findings may not fully reflect broader population-level racial and ethnic distributions. |
| Geography | Participants were enrolled at a single academic cancer center in Boston, MA, USA. Referral patterns to tertiary care institutions may enrich for younger patients, those with access to specialized neuro-oncology care, and individuals able to travel for clinical trial participation. As a result, the study population may not fully reflect the geographic and socioeconomic diversity of patients with HER2+ metastatic breast cancer and BCBM nationally or internationally. |
| Other considerations | This was a single-center phase II trial enrolling patients with active or progressive brain metastases, representing a clinically high-risk population. Eligibility criteria required adequate organ function and good performance status, and 94% of patients had an Eastern Cooperative Oncology Group (ECOG) performance status of 0-1. Patients treated at academic centers may have greater access to multidisciplinary central nervous system (CNS)-directed care, prior lines of HER2-targeted therapy, and clinical trial infrastructure. These factors may limit generalizability to patients treated in community settings. |
| Overall representativeness of this study | Overall, the study population reflects a clinically relevant subset of patients with HER2-positive metastatic breast cancer and active brain metastases treated at tertiary academic centers, particularly with respect to sex distribution and disease characteristics. However, the younger median age, single-institution design, modest racial/ethnic diversity, and small sample size limit broader generalizability. |
