# Supplementary material for: Paxalisib with Trastuzumab for HER2-Positive Metastatic Breast Cancer with Active Brain Metastases: A Phase II Trial with Integrated Plasma and CSF ctDNA Analyses
Source: Cancer Res Commun. 2026 Nov 6;6(11):2619–30. doi: 10.1158/2767-9764.CRC-26-0571 (PMC13741795; doi:10.1158/2767-9764.CRC-26-0571)
Supplement: Supplementary file 6 [file crc-26-0571_supplementary_table_s2_suppst2.docx]

**Supplementary Table S2. Treatment-related adverse events (TRAEs) among patients treated at 45 mg safety run-in (n=5)**

|  | **All grades,**  **n (%)** | **Grade 2,**  **n (%)** | **Grade 3,**  **n (%)** | **Grade 4,**  **n (%)** |
| --- | --- | --- | --- | --- |
| Any event | 4 (80.0) | 1 (20.0) | 2 (40.0) | 1 (20.0) |
| Fatigue | 3 (60.0) | 3 (60.0) |  |  |
| Anorexia | 2 (40.0) | 2 (40.0) |  |  |
| Diarrhea | 2 (40.0) | 2 (40.0) |  |  |
| Hyperglycemia | 2 (40.0) | 1 (20.0) |  | 1 (20.0) |
| Mucositis oral | 2 (40.0) | 1 (20.0) | 1 (20.0) |  |
| Rash acneiform | 2 (40.0) |  | 2 (40.0) |  |
| Abdominal pain | 1 (20.0) | 1 (20.0) |  |  |
| Dehydration | 1 (20.0) |  | 1 (20.0) |  |
| Dizziness | 1 (20.0) | 1 (20.0) |  |  |
| Ejection fraction decreased | 1 (20.0) | 1 (20.0) |  |  |
| Endocrine disorders | 1 (20.0) | 1 (20.0) |  |  |
| Generalized muscle weakness | 1 (20.0) | 1 (20.0) |  |  |
| Hypokalemia | 1 (20.0) |  | 1 (20.0) |  |
| Hypomagnesemia | 1 (20.0) | 1 (20.0) |  |  |
| Hyponatremia | 1 (20.0) |  | 1 (20.0) |  |
| Hypophosphatemia | 1 (20.0) |  | 1 (20.0) |  |
| Nausea | 1 (20.0) |  | 1 (20.0) |  |
| Pruritus | 1 (20.0) | 1 (20.0) |  |  |
| Urinary frequency | 1 (20.0) | 1 (20.0) |  |  |
| Vomiting | 1 (20.0) | 1 (20.0) |  |  |
| Weight loss | 1 (20.0) | 1 (20.0) |  |  |

Grade 1 toxicities were not collected. No grade 5 toxicities were observed. All TRAEs are shown.
